# Junger Mann mit akuter Visusminderung

**DOI:** 10.1007/s00347-021-01484-4

**Published:** 2021-08-27

**Authors:** J. Friedrich, M. Ulbig, M. Maier

**Affiliations:** Klinikum rechts der Isar, Augenklinik, Technische Universität München, Ismaninger Str. 22, 81675 München, Deutschland

## Anamnese

Ein 41-jähriger, männlicher Patient stellte sich mit akuter beidseitiger Visusminderung in unserer Notfallambulanz vor. Er berichtete, eine zentrale graue Wolke zu bemerken, welche am rechten Auge seit 1 Monat und am linken Auge seit 2 Monaten bestehe und nun deutlich zunehme.

Es bestünden weder ophthalmologische noch internistische Vorerkrankungen, die Reiseanamnese war leer.

## Klinischer Befund

Unsere Untersuchungen zeigten einen bestkorrigierten Fernvisus von 0,8 dezimal am rechten Auge und 0,5 dezimal am linken Auge, bei normotensivem Augeninnendruck und reizfreiem vorderem Augenabschnitt.

Der Fundus zeigte am rechten Auge eine randscharf begrenzte und vital gefärbte Papille, die Makula wies einen fovealen Reflex sowie eine flächige Aufhellung auf. Die periphere Netzhaut zeigte einen gelblichen Herd bei 12 Uhr.

Am linken Auge war der Einblick bei hinteren Glaskörperzellen reduziert. Bei 6 Uhr peripher zeigten sich dichte Glaskörpertrübungen. Die Papille erschien auf dem linken Auge ebenfalls randscharf begrenzt und vital gefärbt. Die periphere Netzhaut zeigte sich an beiden Augen zirkulär anliegend.

Nebenbefundlich berichtete der Patient über einen seit ca. 5 Monaten bestehenden rötlich-lividen Fleck von ca. 3 × 3 cm links palmar.

## Diagnostik

### Optische Kohärenztomographie

**Figure 1:**
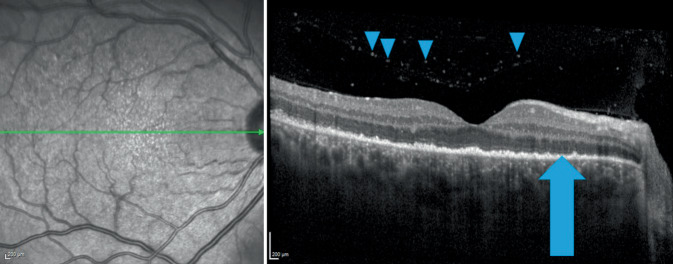

Die optische Kohärenztomographie (OCT) zeigte auf dem rechten mehr als auf dem linken Auge hintere Glaskörperzellen, eine Unregelmäßigkeit der inneren und äußeren Photorezeptorsegmente (IS/OS) sowie des retinalen Pigmentepithels (RPE). Es zeigte sich auf beiden Seiten kein Makulaödem (Abb. 1 und 2).

**Figure 2:**
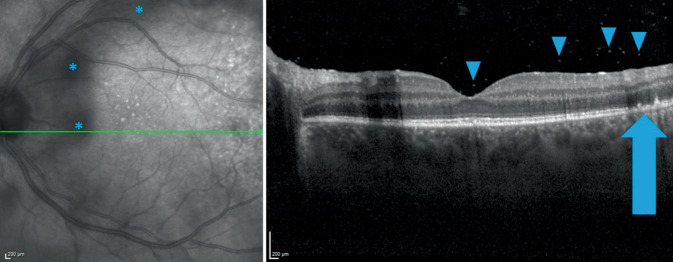

### Fluoreszenzangiographie

**Figure 3:**
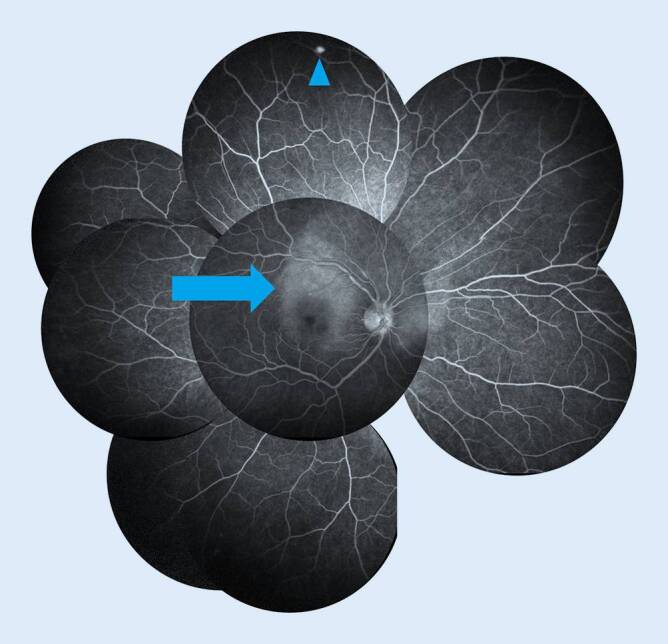

Die Fluoreszenzangiographie (FLA) zeigte auf dem rechten Auge eine regelrechte Gefäßfüllung sowie eine punktförmige Leckage bei 12 Uhr peripher im Bereich des fundoskopisch sichtbaren gelblichen Herdes (Abb. 3). In der Spätphase war am rechten Auge eine flächige Hyperfluoreszenz im Bereich der Makula zu sehen, welche sich über den oberen Gefäßbogen (OGB) und nach nasal der Papille erstreckte. Des Weiteren konnte eine Hyperfluoreszenz der Papille ohne Leckage im Verlauf detektiert werden.

**Figure 4:**
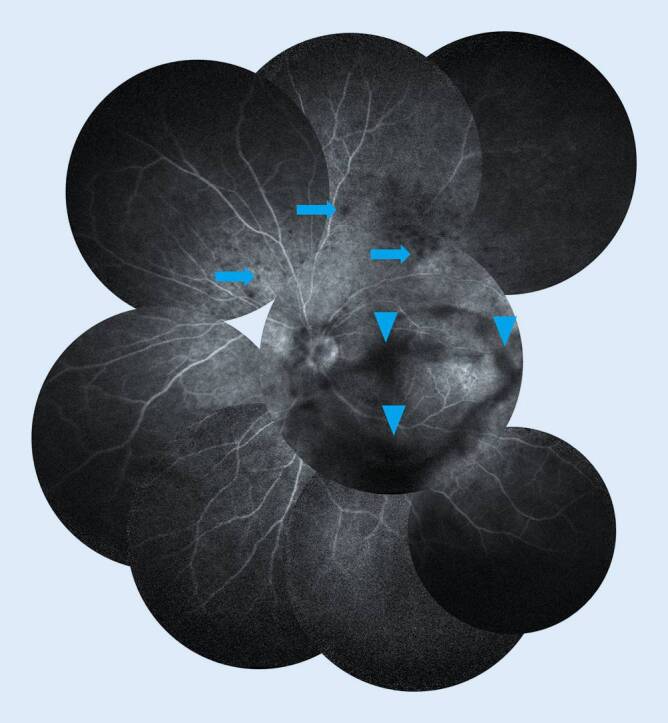

Auf dem linken Auge zeigte die FLA einen regelrechten Farbstoffeinstrom, Abschattungen durch Glaskörpertrübungen, punktförmige Hypofluoreszenzen in der Makula und am OGB sowie eine Papillenhyperfluoreszenz ohne Leckage in der Spätphase (Abb. 4).

## Wie lautet Ihre Diagnose?

## Diagnose und Therapie

Insgesamt zeigte sich fundoskopisch der Befund einer plakoiden Chorioretinitis. In Zusammenschau mit den IS/OS- und den RPE-Unregelmäßigkeiten sowie den hinteren Glaskörperzellen in der OCT und unter Berücksichtigung des jungen Alters des männlichen Patienten bestand klinisch der Verdacht auf eine plakoide Chorioretinitis bei Lues [7, 9].

**Diagnose:** Plakoide Chorioretinitis bei Lues

Wir führten ein mikrobiologisches Screening auf eine Infektion mit *Treponema pallidum, Borrelia burgdorferi, Bartonella henselae, Herpes simplex* und *Varicella zoster* durch.

Außerdem erfolgte ein Laborscreening inklusive Blutbild, Angiotensin-Converting-Enzyme (ACE), Interleukin-2(IL-2)-Rezeptor, C‑reaktives Protein (CRP) sowie rheumatologischen Autoantikörpern (Rheumafaktor, CCP-AK, ANCA, ANA, Sp100-AK).

Die serologische Untersuchung bestätigte die Verdachtsdiagnose einer aktiven, behandlungsbedürftigen Lues (positiver TPPA-Test, positive Luesantikörper vom IgM-Typ, positiver FTA-Test, positive Kardiolipinantikörper).

## Verlauf

Zur Einleitung einer intravenösen antibiotischen Therapie erfolgte eine konsiliarische Vorstellung in der infektiologischen Poliklinik unseres Hauses. Der Patient erhielt dort eine intravenöse antibiotische Therapie mit Ceftriaxon 2 g täglich für insgesamt 10 Tage.

Des Weiteren erfolgte eine Abklärung auf Koinfektionen mit HIV, Hepatitis B und Hepatitis C. Diese konnten serologisch ausgeschlossen werden.

Im Verlauf konnten eine zunehmende Befundbesserung sowie ein signifikanter Abfall des Cardiolipin-Titers von ursprünglich 1:512 auf 1:16 verzeichnet werden.

**Figure 5:**
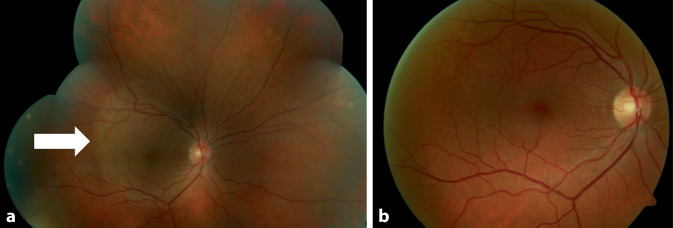

Bei der ambulanten Abschlusskontrolle 10 Wochen nach abgeschlossener Antibiotikatherapie zeigten sich ein bestkorrigierter Fernvisus von 1,0 dezimal beidseits sowie ein strukturophthalmologisch regelrechter Befund. Die OCT zeigte ebenfalls eine komplette Remission (Abb. 5 und 6).

**Figure 6:**
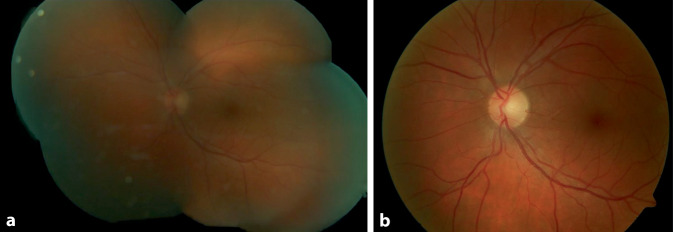

## Pathogenese

Die Lues (Synonym: Syphilis) ist eine meldepflichtige, sexuell übertragbare Infektionserkrankung, welche durch das gramnegative Bakterium *Treponema pallidum* aus der Familie der Spirochäten ausgelöst wird [2, 4].

Seit 2010 zeigt sich ein Anstieg der gemeldeten Luesfälle in Deutschland, wobei der Großteil der Patienten Männer in der Altersgruppe von 30 bis 39 Jahren ist [1, 4].

Bei etwa der Hälfte der verzeichneten Fälle besteht eine Koinfektion mit dem humanen Immundefizienzvirus (HIV). Bei jedem Patienten mit bestätigter Luesinfektion sollte daher auch auf eine HIV-Infektion untersucht werden [8].

Nach einer Inkubationszeit von durchschnittlich 14 bis 24 Tagen kommt es zunächst zu einem hochinfektiösen Stadium I, wobei etwa die Hälfte der Patienten Symptome in Form einer derben Induration an der Eintrittspforte des Erregers, das sog. Ulkus durum zeigen. Etwa 4 bis 10 Wochen nach der Infektion kommt es zu einer hämatogenen und lymphogenen Aussaat mit generalisierten Krankheitserscheinungen, dem weiterhin infektiösen Stadium II. Die Frühsyphilis umfasst das Stadium I und II und ist für eine Zeitspanne von bis zu 1 Jahr nach der Infektion definiert. Zur Spätsyphilis zählen das Stadium III, in welchem kardiovaskuläre Komplikationen auftreten können, sowie das Stadium IV, welches auch als Neurosyphilis bezeichnet wird, da es hier zu einer ZNS-Beteiligung kommen kann [4].

## Diagnostik und Therapie

Laut der aktuellen Leitlinie der Arbeitsgemeinschaft der Wissenschaftlichen Medizinischen Fachgesellschaften (AWMF) sollen bei Verdacht auf eine Luesinfektion zunächst Suchtests durchgeführt werden (TPPA- oder TPHA-Test). Bei positivem Ergebnis soll ein Bestätigungstest durchgeführt werden (FTA-Abs-Test, *Treponema-pallidum*-IgM-ELISA oder IgM-Western-Blot). Zur Verlaufsbeurteilung bietet sich als Aktivitätsparameter eine quantitative Kardiolipinreaktionstestung an. Bei Verdacht auf eine Neurolues soll eine Liquordiagnostik durchgeführt werden [3].

Eine ophthalmologische Beteiligung soll immer wie eine Neurolues behandelt werden [1, 8].

Eine Augenbeteiligung kann jedoch in jedem Stadium einer Lueserkrankung auftreten und nahezu jeden Teil des Auges betreffen. Neben Skleritis, Sklerokeratitis und Iridozyklitis kann sich eine okuläre Lues auch in Form einer anterioren, intermediären oder posterioren Uveitis sowie in Form einer Panuveitis äußern. Des Weiteren kann es zu einer Papillitis oder zu einem Arterienastverschluss kommen, wobei jedoch die häufigste ophthalmologische Manifestation der okulären Lues die zentrale plakoide Chorioretinopathie darstellt [5, 6].

Das therapeutische Mittel der Wahl bei einer aktiven Luesinfektion ist für jedes Stadium die antibiotische Therapie mit Penicillin über mindestens 10 Tage. Bisher sind keine Resistenzen des Erregers gegen Penicillin bekannt [4].

Im Detail bedeutet dies nach der aktuellen AWMF-Leitlinie für die Neurolues die systemische, intravenöse Therapie mit Penicillin G 4‑mal 6 Mio. IE pro Tag, 5‑ mal 5 Mio. IE pro Tag oder 3‑mal 10 Mio. IE pro Tag (entspricht 3–4 Mio. IE alle 4 h) über 14 Tage. Alternativ wird eine systemische Therapie mit Ceftriaxon 2 g täglich (entspricht 2 g alle 24 h) intravenös über 14 Tage empfohlen. Als Mittel der dritten Wahl gibt es eine Empfehlung für eine systemische Therapie mit Doxycyclin-Tabletten 2‑mal 200 mg pro Tag (entspricht 200 mg alle 12 h) für 28 Tage [3].

## Fazit für die Praxis

- Seit 2010 zeigt sich eine Zunahme der gemeldeten Luesfälle in Deutschland.
- Bei plakoider Chorioretinitis soll immer auch an Lues gedacht werden.
- Eine Augenbeteiligung ist in jedem Luesstadium möglich.
- Jede ophthalmologische Luesmanifestation soll als Neurolues behandelt werden.
- Eine Anbindung an ein infektiologisches Zentrum ist sinnvoll.
- Eine Abklärung von Koinfektionen wie HIV, Hepatitis B und Hepatitis C soll immer erfolgen.
- Die intravenöse antibiotische Therapie bei Lues-assoziierter Uveitis ist notwendig.
- Eine komplette Remission sowie eine vollständige Visuserholung durch eine adäquate Therapie ist möglich.
